# Evaluation of surface type and time of day on agility course performance

**DOI:** 10.3389/fvets.2024.1415634

**Published:** 2024-06-26

**Authors:** Arielle Pechette Markley, Nina R. Kieves, Linda Blake Rivas, Abigail B. Shoben

**Affiliations:** ^1^Department of Veterinary Clinical Sciences, College of Veterinary Medicine, The Ohio State University, Columbus, OH, United States; ^2^Red Sage Integrative Veterinary Partners, Fort Collins, CO, United States; ^3^Sidney Kimmel Medical College, Thomas Jefferson University, Philadelphia, PA, United States; ^4^Division of Biostatistics, College of Public Health, The Ohio State University, Columbus, OH, United States

**Keywords:** agility, surface, speed, sports performance, injury, canine sports medicine, biomechanics, canine agility

## Abstract

**Introduction:**

Canine agility competitions are performed on a variety of surfaces. In the equine and human literature, surface type has been associated with speed, performance, and injury risk. The aim of this study was to evaluate the effect of general surface type and time of day on calculated speed (yards per second over a measured course distance) and course performance during the UKI Agility International (UKI) U.S. Open. We hypothesized that surface type would affect calculated speed, with sand being the slowest.

**Materials and methods:**

Data on course performance from the 2021 and 2022 events were obtained directly from UKI. The officiating judge measured course length, automatic timers recorded dogs’ course times, and speeds were calculated from these values. Three surfaces (dirt, grass, and sand) were compared across three categories of courses (jumpers, standard, and speedstakes). Differences in calculated speeds and qualifying rates were estimated using generalized estimating equations (GEE) to account for multiple runs by the same handler.

**Results:**

Among jumpers courses, those run on sand in 2021 were markedly slower than those run on dirt. Grass and dirt were more similar in terms of average calculated speed, though some courses run on grass were significantly faster than courses run on dirt and vice versa. Time of day effects observed were inconsistent, with more variability observed for dirt and sand than for grass.

**Discussion:**

There was a notable variation in calculate speed based on surface with sand being slowest, likely due to the increased energy cost required to run on sand due to its high compliance. Calculated speeds on grass and dirt appeared generally similar, but there was substantial variability of calculated speed among various courses, making comparison of surface effects challenging. Variables within the surface itself (such as compaction level and moisture content) likely play a role in the effects of surface on speed and performance. This study provides insight into the complexity of surface effects on performance in agility dogs and highlights the need for canine-specific surface studies on the effect of surface variables and how these relate to risk of development of musculoskeletal injuries.

## 1 Introduction

Canine agility is a popular performance sport where dogs navigate a pre-set course of obstacles with the winner completing the course in the fastest time with the fewest number of errors. Courses include jump obstacles, tunnels, and contact obstacles such as the A-frame, seesaw, and dog walk. The variety of obstacles and course layouts present ever-changing physical demands on the dog. Combined with high speed and technicality of some courses, there is the potential of both repetitive stress injuries and acute trauma. With the high injury rate of up to 41.7% (1), there is increasing interest in determining risk factors for injury in order to better inform prevention and treatment strategies.

Canine agility performance is multidimensional, as both speed and precision are critical to success. A perfect agility performance, also commonly called a “clean run” or “qualifying run,” is defined as a dog who traverses all obstacles in the correct order within standard course time and without accruing any errors, or “faults.” There are a variety of common faults: knocking down jump bars; jumping off of or leaving contact obstacles prematurely; and refusing obstacles by spinning in front of, hesitating before, or turning away from obstacles rather than taking them when directed (2). If a dog takes an obstacle out of order or accumulates too many faults they are said to be “eliminated.” Automatic timers are used at the first and last obstacle to record the total time in seconds that it takes for dogs to complete the course. This also allows calculation of average course speed (yards per second/YPS) based on judges’ course distance measurements. Rules about what constitutes faults and eliminations, and the number of faults a given error incurs are specific to the agility organization sanctioning the event. Rules about faults may also be specific to the event itself (i.e., a local competition versus a national competition) (2).

Agility dogs often compete on a variety of surfaces, including dirt, artificial turf, sand, grass, and rubber matting. The specific surface composition determines the surface’s mechanical behaviors, such as cohesion, shear, friction, and vertical displacement while undergoing load or shear forces (3–10). Extrinsic factors such as temperature, moisture level, and how the surface is maintained also affect its mechanical behaviors (11–15). The biomechanical demands on the dog vary based on the body’s interaction with those specific surface properties (3, 16–23). There have been numerous studies evaluating the biomechanical interaction between specific surfaces and human and equine athletes in a variety of contexts (24–30). No studies have evaluated the biomechanical effects of surface composition interaction in dogs. In the equine and human literature, surface has been shown to be associated with injury risk (31–36). For example, Thoroughbred racehorses have a 32% higher risk of sustaining a fracture when racing on a dirt surface compared to a synthetic surface (37). The types of injuries seen are influenced by the surface composition and specific sport interaction (36, 38). Surface has also been implicated in injury in racing Greyhounds (39). While retrospective surveys have tried to evaluate associations between surface and agility dog injury (40), there is little evidence evaluating the effect of surface on agility performance.

The aim of this study was to evaluate the effect of general surface and time of day on calculated speed and course performance during the UKI Agility International (UKI) U.S. Open, a large national multi-day event with multiple runs per day completed on a variety of surfaces. We hypothesized that surface type would affect calculated speed, with sand being the slowest surface. We also hypothesized that the time of day would affect calculated speed, with lower calculated speeds on sand early in the day due to fresh harrowing conditions, and faster calculated speeds later in the day due to more compacted surface conditions.

## 2 Materials and methods

Data from all runs of the 2021 and 2022 UKI U.S. Open were obtained from UKI directly. The results spreadsheet acquired from UKI consisted of one row per run and included handler name, dog name, competition jump height category, course name, time (measured by the automatic timers), faults, and an indicator of if the team had been eliminated during the run. Additional information about the event was obtained from information published on the UKI website at the time of the events.

The two main outcomes (qualifying run rate and calculated speed) were inferred from this information. An individual dog “qualified” on a specific course if it had a recorded time, had zero faults, and had not been eliminated (i.e., it had a clean run). Among dogs with clean runs, speed (YPS) was calculated from the recorded time and the reported total course length. The total course length was measured by the officiating judge following standard UKI practice of measuring the shortest distance in yards between each obstacle in sequence (2). The sum of these between obstacle distances plus the length of each obstacle dogs must traverse was recorded as the total course length.

The 2021 and 2022 events were held at the Jacksonville Equestrian Center in Jacksonville Florida. Information about the type of surface in each ring was obtained directly from the venue. A general overview of the rings and surfaces is shown in Table 1. Four dirt rings were in use both years; the surface composition in that area was local Florida soil (dirt), with no specific types or subtypes noted. Two of these rings were in a climate controlled, covered area, and two of these rings were in a covered area that was not climate controlled. Two rings of grass in an outdoor, uncovered area were used both years; this surface was predominantly Bermuda grass, with small amounts of other subtypes of local Florida grass mixed in. In 2021, two additional rings were run in an outdoor arena that consisted of sand/fiber footing.

**Table 1:** Description of ring surfaces and environment at the 2021 and 2022 U.S. Opens.

Surface	Surface notes	Ring numbers/notes	Environment
Dirt	Local Florida soil	1&2	Climate controlled, covered
		3&4	Outdoor, covered
Grass	Predominantly Bermuda grass	5&6	Outdoor, uncovered
Sand	Sand/fiber footing	7&8 (only used in 2021)	Outdoor, uncovered

Surface maintenance was performed daily, in the morning prior to any runs. The ring preparation involved harrowing using Kiser Dragmasters, Reveal 4-n-1, and Carolina DragNfly (designed for sand/fiber rings), and two rollers that help to compact the moisture from when the surface is watered at night. The nightly maintenance involved adding water to the surface to increase the moisture of the substrate. There was no specific amount of water used and the amount added was based on operator discretion in relation to the weather and humidity at the time. During the competition day, the surface was not refreshed at any time during the daytime, but when rings were combined and reset before evening event finals, they would perform a refresh of the surface.

Both 2021 and 2022 UKI U.S. Opens could be entered by any dog and handler team registered with UKI. Competitors could choose which of several events to enter (e.g., Biathlon, Masters series, and Speedstakes). Some events consisted of multiple courses, and some courses required a certain level of performance in an earlier course to participate (e.g., speedstakes final took only dogs who achieved a top score in the speedstakes semi-final). Courses were categorized into three classes: standard classes that include all obstacles including jumps and contact obstacles; jumpers classes that include jumps, tunnels, and weaves, but no contact obstacles; and speedstakes classes that include only regular bar jumps and tunnels (41).

During both events, competitors were randomly assigned to “rotation groups,” which meant that the time of day a particular dog was running a particular course was a function of their randomly assigned group. Signalment information on the dogs competing was not available; however, information about the height of the dog was inferred from their competition jump height. Dogs were assigned to a competition jump height category based on their height at the withers; handlers could optionally elect to jump one height category lower for any reason (“select class”).

We evaluated difference in qualifying run rates and average calculated speed (YPS) among classes that had some variation in surface; the jumpers classes in 2021 (5 courses, 1 on dirt, 2 on grass, and 2 on sand), the jumpers classes in 2022 (4 courses, 3 on dirt, 1 on grass), and the speedstakes semi-final course in 2022 that was run on both grass on dirt. The speedstakes semi-final course in 2022 is a unique comparison as the course was the same for both the grass and dirt surface. The other comparisons are among courses in the same class (jumpers) but varied in course design.

Models to estimate these differences used all available runs from each year and adjusted for specific course, height category and if the dog was running in the select class. All models used the method of generalized estimating equations (GEE) with robust standard errors adjusted for clustering among runs from the same handler.

To evaluate the potential impact of time of day on calculated speed and qualifying rate by general surface type, we fit models examining the impact of rotation group on calculated speed (YPS) and qualifying rate using GEE. These models were fit separately for each of the three classes (standard, jumpers, and speedstakes) by year and allowed the impact of rotation group to vary by surface type within each class.

All analyses were performed using Stata version 15.1. All *p*-values are presented unadjusted for multiple comparisons, except within year and class, we indicated pairwise comparisons that were significant after Holm correction. We considered the analysis of differences in speed by surface to be the primary analyses.

## 3 Results

For the 2021 event, there were 458 handlers running 706 unique dogs across the entire event. Most handlers ran one (*n* = 267, 58%) or two (*n* = 149, 33%) dogs, with 9% of handlers (*n* = 42) running three or more dogs. The 2022 event was somewhat larger with 553 unique handlers running 870 unique dogs. The percentage of handlers running one (*n* = 298, 54%), two (*n* = 206, 37%), or three or more dogs (*n* = 49, 9%) was similar to 2021.

In 2021, a total of 2,216 jumpers runs were recorded across five different courses (593 on dirt on one course, 931 across two courses on grass, and 692 across two courses on sand). In 2022, a total of 2,262 jumpers runs were recorded across four different courses (1,790 across three courses on dirt and 472 on one course on grass). Also in 2022, the same speedstakes course was run on both grass (538 runs) and dirt (275 runs). Additional runs on dirt were evaluated in both 2021 and 2022 for standard and speedstakes classes (Table 2). In both 2021 and 2022, qualifying run rates and average calculated speed (YPS) varied by the type of course and the individual course itself (Table 2), with higher calculated speeds observed for the speedstakes type courses (only jumps and tunnels) and lower calculated speeds and somewhat less variable calculated speeds for standard courses that included contact obstacles.

**Table 2:** Percentage of qualifying runs and mean calculated speed (YPS) speeds for all courses run in the 2021 and 2022 U.S. Open.

Year – class	Surface	*N* runs	*N* clean (%)	Mean YPS (*sd*)
**Jumpers classes – 2021**				
2021 – Biathlon Jumping	Dirt	593	95 (16.0%)	5.5 (0.8)
2021 – Masters Final Jumping	Grass	405	92 (22.7%)	5.1 (0.6)
2021 – Winner Take All	Grass	526	109 (20.7%)	6.0 (0.7)
2021 – Last Chance Masters Jumping	Sand	298	58 (19.5%)	5.4 (0.6)
2021 – UKI Nationals Round 1	Sand	394	63 (16.0%)	5.0 (0.5)
**Jumpers classes – 2022**				
2022 – Masters Final Jumping	Grass	472	50 (10.6%)	5.7 (1.0)
2022 – Winner Take All	Dirt	695	261 (37.6%)	6.3 (0.8)
2022 – Biathlon Jumping	Dirt	692	161 (23.3%)	5.6 (0.7)
2022 – UKI Nationals Round 1	Dirt	403	49 (12.2%)	5.3 (0.7)
**Standard classes – 2021**				
2021 – Last Chance Masters Agility	Dirt	310	32 (10.3%)	5.3 (0.7)
2021 – Masters Final Agility	Dirt	413	87 (21.1%)	4.9 (0.6)
2021 – UKI Nationals Round 2	Dirt	339	76 (22.4%)	5.0 (0.6)
**Standard classes – 2022**				
2022 – Last Chance Masters Agility	Dirt	348	19 (5.5%)	5.2 (0.6)
2022 – Masters Final Agility	Dirt	499	108 (21.6%)	5.1 (0.8)
2022 – UKI Nationals Round 2	Dirt	425	61 (14.4%)	5.0 (0.8)
2022 – US Open Agility	Dirt	677	90 (13.3%)	5.2 (0.6)
**Speedstakes classes – 2021**				
2021 – Power and Speed (speed portion)	Dirt	414	91 (22.0%)*	5.4 (0.5)
2021 – Speedstakes Round 1	Dirt	665	235 (35.3%)	5.7 (0.7)
**Speedstakes classes – 2022**				
2022 – Speedstakes Round 1 (group A)	Dirt	275	74 (26.9%)	5.5 (0.7)
2022 – Speedstakes Round 1 (group B)	Grass	538	76 (14.1%)	6.3 (0.8)

For each course, the number of dogs who started the course (*N* runs) is reported, as well as the number who ran the course as described without any errors or faults (*N* clean). From these, the percentage of dogs who ran clean (%) is calculated and reported. Among the dogs who ran clean, the mean calculated speed as Yards Per Second (YPS) is reported as well as the standard deviation (*sd*) of these values. *Of all dogs that started the Power & Speed course, not all errors made during the speed portion.

### 3.1 Surface effects on calculated speed

**Figure 1:**
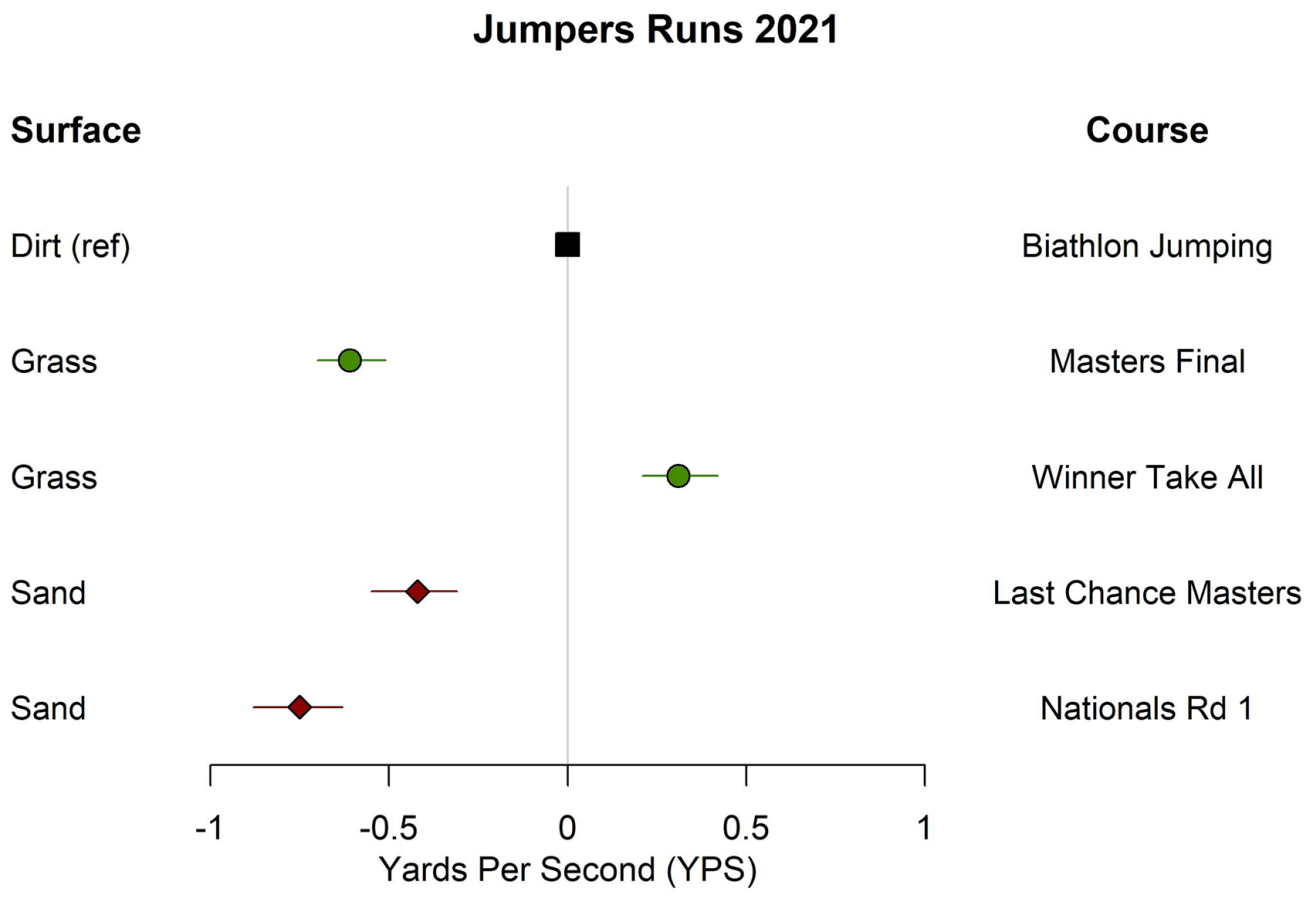
Estimated differences in calculated speed for jumpers courses run on different surfaces at the 2021 U.S. open. Estimates are adjusted for height category and if the dog was running select.

In 2021, the mean calculated speed for both jumpers courses run on sand was significantly lower than mean calculated speed for the jumpers course run on dirt (0.42 and 0.75 YPS slower; Table 3 and Figure 1). The two sand courses were also significantly slower than one of the courses run on grass, but they were closer in calculated speed to the other grass course, with one course run on sand faster and the other slower than the slowest grass course. There was significant variation in calculated speed between the two jumpers courses run on grass, where one course was significantly faster than dirt and the other was significantly slower (Table 3).

**Table 3:** Estimated differences in calculated speed for jumpers and speedstakes courses run on different surfaces.

	Mean difference in speed (YPS, 95% CI)	Significant pairwise differences*
**2021 Jumpers courses**		
^a^Dirt (Biathlon Jumping)	(Ref)	All significant (10 total)
^b^Grass1 (Masters Final Jumping)	−0.61 (−0.70, −0.51)	
^c^Grass2 (Winner Take All)	0.31 (0.21, 0.42)	
^d^Sand1 (Last Chance Masters)	−0.42 (−0.55, −0.30)	
^e^Sand2 (UKI Nationals Rd1)	−0.75 (−0.88, −0.63)	
**2022 Speedstakes Round 1**		
Dirt	(Ref)	One (*p* < 0.001)
Grass	0.35 (0.22, 0.48)	
**2022 Jumpers courses**		
^a^Grass (Masters Final Jumping)	(Ref)	All significant (6 total)
^b^Dirt1 (Biathlon Jumping)	−0.15 (−0.27, −0.04)	
^c^Dirt2 (Winner Take All)	0.45 (0.34, 0.56)	
^d^Dirt3 (UKI Nationals Rd1)	−0.38 (−0.52, −0.24)	

YPS, Yards Per Second; negative values indicate slower YPS relative to the reference category. 95% CI, 95% confidence interval, estimated from a model adjusted for height category, if the dog was running select, and accounting for clustering by handler. *Pairwise comparisons done within each year and type of course combination and comparisons that were significant after the Holm correction are identified. ^a–e^ are used as superscripts to identify pairwise differences.

**Figure 2:**
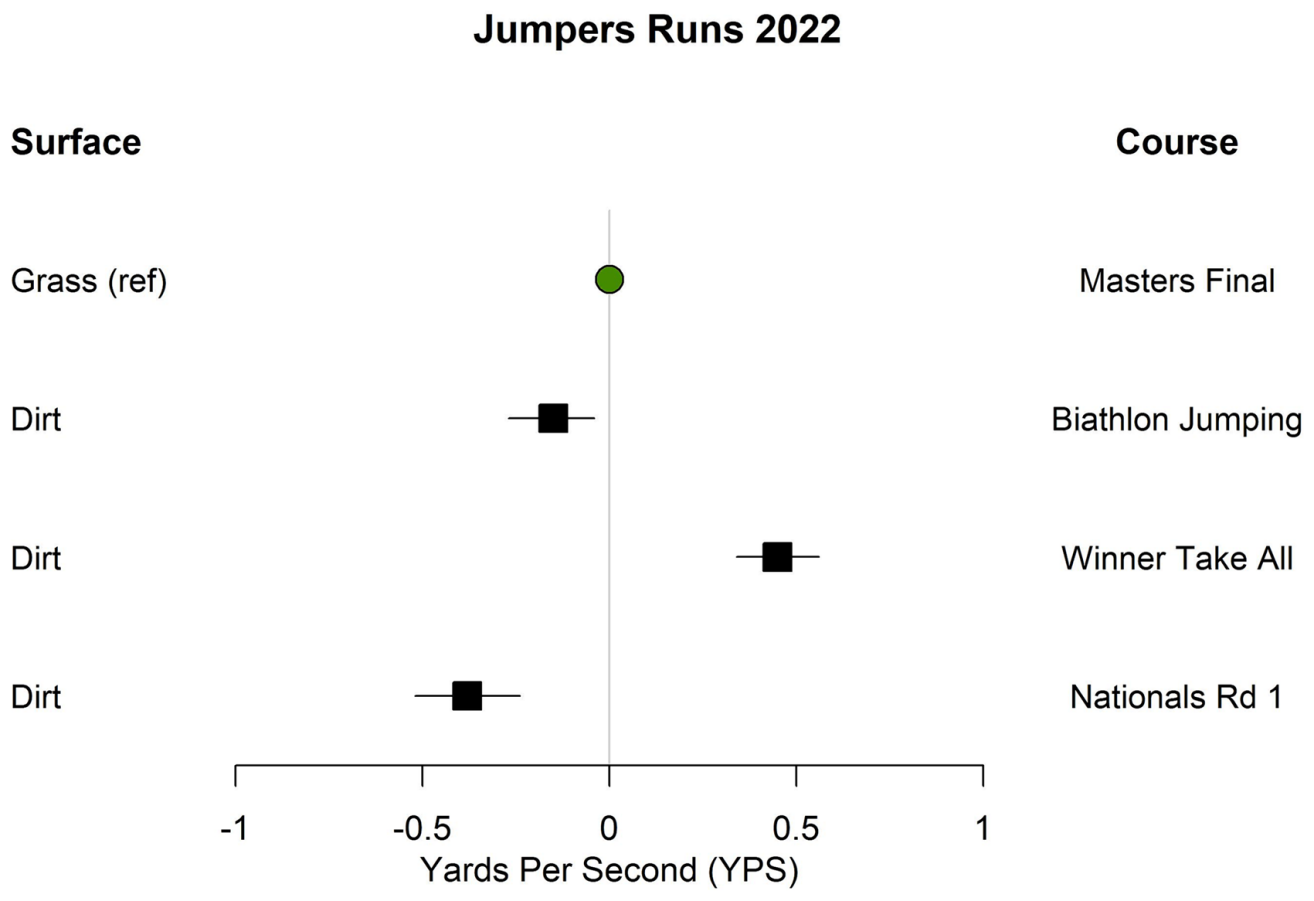
Estimated differences in calculated speed for jumpers courses run on different surfaces at the 2022 U.S. open. Estimates are adjusted for height category and if the dog was running select.

In 2022, there was significant variability in the calculated speeds among the three jumpers courses run on dirt and the single jumpers course run on grass (Table 3 and Figure 2). The course run on grass was significantly faster than two of the three jumpers courses run on dirt, but was significantly slower than the third jumpers course run on dirt. The same speedstakes course was run on both grass and dirt in 2022; the mean calculated speed was significantly higher on grass than dirt (0.35 YPS higher, 95% CI: 0.22 to 0.48 higher; Table 3).

### 3.2 Time of day effects on calculated speed

**Figure 3:**
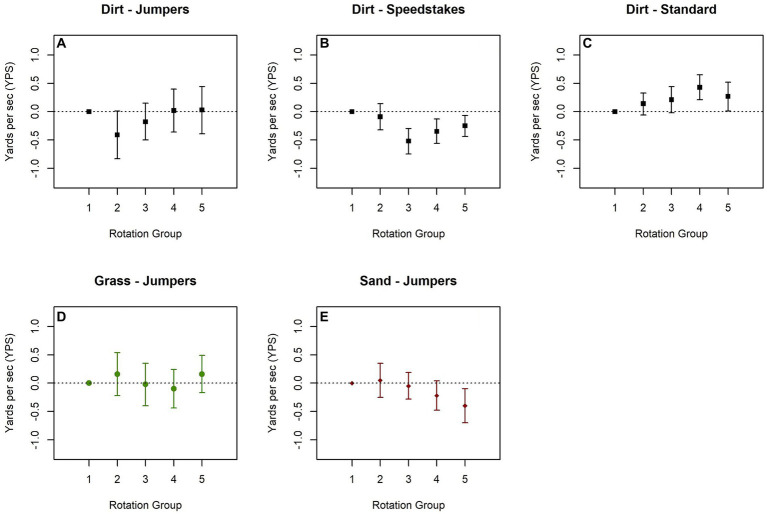
Estimated difference in calculated speed difference by order among classes run on dirt **(A–C)**, grass **(D)**, and sand **(E)** from the 2021 U.S. Open. Models are adjusted for height class and if dog is select. Plots show the trend by rotation group with the earliest group (1) used as the reference.

In 2021, effects of time of day on calculated speed on dirt were inconsistent by class type (Figures 3A–C and Supplementary Table S1). In jumpers classes, the lowest calculated speeds were observed midday, with higher calculated speeds observed during earlier and later rotations. In speedstakes classes, lower calculated speeds were observed later in the day, while for standard classes, higher calculated speeds were observed later in the day. There was very little variation observed by time of day for calculated speed on grass (Figure 3D and Supplementary Table S1). In contrast on sand, slower calculated speeds were observed later in the day (Figure 3E and Supplementary Table S1).

**Figure 4:**
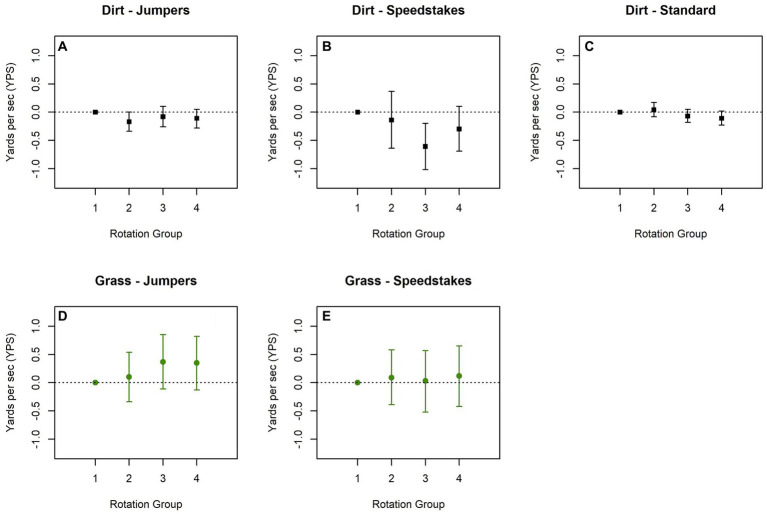
Estimated difference in calculated speed difference by order among classes run on dirt **(A–C)** and grass **(D, E)** from the 2022 U.S. Open. Models are adjusted for height class and if dog is select. Plots show the trend by rotation group with the earliest group (rotations 1 and 2) used as the reference.

In 2022, the effects of time of day by class type on dirt were again inconsistent (Figures 4A–C and Supplementary Table S2). There was low variability observed for the jumpers and standard classes run on dirt. For speedstakes, slower calculated speeds were observed later in the day, similar to 2021. On grass in 2022, average calculated speed was slowest during the earliest rotations in the jumpers class, but variability was high, and there was very little variability in the speedstakes class (Figures 4D,E and Supplementary Table S2).

### 3.3 Effects on qualifying rates

In 2022, qualifying rates for the one jumpers course run on grass were significantly lower than two of the three jumpers courses run on dirt, and slightly lower (although not statistically different) than the third (Table 4). Similarly, dogs were significantly less likely to qualify on the same speedstakes course in 2022 if running on grass than dirt (0.092 lower probability of qualifying on grass, Table 4). However, no significant differences were observed related to the probability of qualifying among the five jumpers courses run in 2021, and the direction of the estimated effects suggested dogs were more likely to qualify running on grass or sand compared to dirt (Table 4). No large differences in qualifying rates by time of day were observed in either 2021 or 2022 (Supplementary Tables S3, S4).

**Table 4:** Estimated differences in probability of qualifying for jumpers and speedstakes courses run on different surfaces.

	Mean difference in probability of qualifying (95% CI)	Significant pairwise differences*
**2021 Jumpers courses**		
^a^Dirt (Biathlon Jumping)		None (10 total)
^b^Grass1 (Masters Final Jumping)	0.045 (−0.003, 0.924)	
^c^Grass2 (Winner Take All)	0.056 (0.014, 0.097)	
^d^Sand1 (Last Chance Masters)	0.057 (0.003, 0.111)	
^e^Sand2 (UKI Nationals Rd1)	0.014 (−0.029, 0.056)	
**2022 Speedstakes Round 1**		
Dirt	(Ref)	One (*p* < 0.001)
Grass	−0.092 (−0.144, −0.040)	
**2022 Jumpers courses**		
^a^Grass (Masters Final Jumping)	(Ref)	All significant except a vs. d
^b^Dirt1 (Biathlon Jumping)	0.134 (0.096, 0.172)	
^c^Dirt2 (Winner Take All)	0.282 (0.239, 0.325)	
^d^Dirt3 (UKI Nationals Rd1)	0.032 (−0.004, 0.069)	

Positive differences in probability of qualifying indicate larger probability of qualifying relative to the reference category. 95% CI, 95% confidence interval, estimated from a model adjusted for height category, if the dog was running select, and accounting for clustering by handler. *Pairwise comparisons done within each year and type of course combination and comparisons that were significant after the Holm correction are identified. ^a–e^ are used as superscripts to identify pairwise differences.

## 4 Discussion

### 4.1 Surface effects on calculated speed

As was hypothesized, there was a notable variation in calculated speed based on surface type with sand appearing to be a slower surface. Sand is generally a softer surface and requires a higher energy cost during running compared to running on harder surfaces (42). This higher energy cost is due to an increase in muscle activation resulting from increased joint range of motion and a decrease in muscle-tendon efficiency (42, 43). Due to the high compliance of sand, the surface also acts as a damper and reduces take-off velocity (43). The combination of these biomechanical interactions with sand, result in it being a slower surface compared to harder surfaces (44, 45).

It is important to note that the compositions of equine sand arenas, such as the one utilized at this event, are different from the sand surfaces utilized in most human athletic events, such as beach volleyball. Most human sand studies, whether studies evaluating running on sand or studies evaluating athletic events on sand, take place on 100% sand surfaces. There are still composition and biomechanical differences in these human-utilized sand surfaces based on particle size, specific mineral content, and whether the sand is wet or dry (18). The 100% sand surfaces in human studies more closely mirror those studies in harness trotters (18, 46). The sand-like surfaces in equine arenas are considered “synthetic surfaces” because they are typically composite surfaces of sand/fiber or sand/rubber. The other components are added to sand to decrease stiffness, improve shear strength and decrease compaction of the sand (36, 47). The variation in type and size of the fibers and the type and size of the rubber affect equine biomechanics in different ways (36). It would be expected that these surface component variations would also affect canine biomechanics, though no studies have been performed to evaluate these effects.

Calculated average speeds in YPS on grass and dirt appeared generally similar, with some courses on dirt having higher calculated speeds than courses run on grass, but some courses on grass having higher calculated speeds than dirt. There was also a substantial amount of variability in calculated speed among the various jumpers courses, making comparison of surface effects challenging. The 2022 speedstakes course that was run on both dirt and grass provides a head-to-head comparison of calculated speed, with higher calculated speeds observed for grass. However, the substantially lower qualifying rate on grass raises the potential that less competitive (slower) dogs were less likely to qualify on grass, making it appear that grass was faster than dirt.

Dirt and grass have different mechanical properties that would be expected to have effects on speed (48). It has also been shown that the incidence of fatal racing injuries in Thoroughbred racehorses is higher on dirt tracks than grass tracks (49), indicating a significant difference in biomechanical effects. However, in many human sports, grass (natural turf) fields have fallen out of favor in place of artificial turf due to the higher risk of injuries and concussions associated with playing on grass compared to artificial turf (32, 50). There are many other variables that affect the comparison between grass and dirt courses in this study, as well as across sports and species. There are significant effects of moisture content, temperature, and maintenance on grass and dirt surfaces. In Thoroughbred racing, it has been shown that speeds are higher with dry track conditions due to increased surface firmness, so the fluctuation in moisture content of the dirt and grass surfaces throughout the day could be confounding factors for course speed (48). Temperature has also been shown to affect surface mechanics as well as speed during racing (14, 51). Surfaces with higher temperatures have been shown to have reduced vertical displacement of the surface and reduced vertical impulse, thereby potentially increasing speed (51). In this study, the dirt surfaces were all covered (and half were in a climate-controlled building), and the grass surfaces were exposed, which leads to the potential for temperature to be a confounding variable for the comparison of dirt versus grass in this study.

### 4.2 Time of day effects on calculated speed

The type and schedule of arena surface maintenance varies by agility event. Surface maintenance for dirt and synthetic surfaces can include adjusting the moisture content and adjusting the depth of the top layer of the material, also known as the uncompacted layer. The moisture content is adjusted through watering the material. The depth of the uncompacted layer can be increased by harrowing, i.e., using specialized equipment to rake/groom the surface thereby loosening the material, or the depth can be decreased by compacting the surface using rollers. These UKI events primarily performed harrowing in the morning before the event started and watering with compacting at night. The surface was not maintained during the day and therefore, it can be assumed that as dogs ran on the surface, the surface properties changed throughout the day in the absence of maintenance. For the synthetic and dirt surfaces, the cushion depth is going to be greatest in the morning after harrowing. This could potentially result in slower speeds and increased energy expenditure, thereby resulting in lower qualifying rates if dogs were more likely to fault due to the cushion depth (51). Since the surfaces were not harrowed throughout the day, it would be expected that as the surface compacted that the vertical displacement would decrease, resulting in faster speeds, and also resulting in higher impact forces and potentially increased injury risk (51).

However, when evaluating the time-of-day effects from this event, the results were not consistent with these expectations. There was a noticeable order effect on dirt for speedstakes, where later runs were actually slower (particularly midday) in both 2021 and 2022, which is opposite of the expected effect of surface compaction. This same pattern was not consistently observed for dirt on standard courses. There was a small trend in a similar direction for standard runs in 2022, but a larger trend in opposite direction for standard runs in 2021. Sand had lower calculated speeds late in the day (jumpers from 2021) and dogs were less likely to qualify on sand early in the day. Therefore, it is likely that other surface variables, such as moisture content and temperature, could be affecting these results. The other consideration is the effect of ruts created by the dogs running the same course throughout the day. While there are no studies that have evaluated the effects of ruts on speed, performance, or injury in agility dogs, it is possible that the more compliant the surface, the more likely there are to be ruts created over time. These ruts could cause dogs to slow down or even fault depending on how the line of the dog corresponds to the ruts. It is also possible that smaller dogs may be more affected by these ruts than larger dogs. The impact of ruts on equine performance is likely less due to the regular harrowing and larger size of the horse compared to the dog. Regular surface maintenance and harrowing of equine arenas are recommended during equine performance training and events in order to prevent surface compaction and reduce risk of injury but it is unknown if a similar recommendation should be made for agility dogs (51).

Variation in grass surface is also likely to influence speed, performance and potentially injury rates. While grass surfaces are less prone to the effects of compaction as synthetic or dirt surfaces, and therefore require less maintenance throughout the day, they may be more prone to environmental (temperature and weather) and moisture effects. In areas where humidity is high, like Florida, grass will often be wet in the morning, potentially resulting in more slipping during jumping and tight turns that could affect both course speeds and qualifying rates. However, there was very little difference in calculated speed or qualifying rate by time of day for all courses run on grass. This may indicate that grass is a more consistent surface, regardless of environmental effects, or it is possible that the observed days had limited variation in environmental effects.

### 4.3 Effects on qualifying rates

In 2021, dogs were somewhat more likely to have qualifying jumpers runs on grass and sand than on dirt. In 2022, dogs were far less likely to qualify on grass than on dirt. It is unknown whether the differences in qualifying rates were due directly to surface effects on speed and biomechanics, or whether they were due to differences in course design, the specific dogs running those courses, surface mechanics factors, or environmental factors. It is possible that the specific combination of surface and type of course, whether it is a more technical course with tighter turns and more complex handling versus a wide-open running course, could also influence qualifying rates and speeds. For example, it is possible that even though sand is a generally slower surface, there may not be as much of an effect on performance for wide open running courses as the more technical courses where the sand would have a larger effect on the ability to accelerate after the greater and more frequent decelerations required to navigate a technical course.

### 4.4 Limitations

One factor that makes evaluating agility performance complex and challenging, particularly with regards to the effects of surface, is the handler component. Since agility is a handler-directed sport, the biomechanical effects of the surface not only affect the dog, but also the handler. The effects of surface on the handler may make it more or less difficult for the handlers to navigate the course, thereby affecting the timing of directions and cues and causing variation in the speed and accuracy of their dog’s performance. This effect is likely more noticeable in technical courses (e.g., biathlon) and less noticeable in wide open courses where dogs are likely to make accurate assumptions about where to go without handler cues.

Limitations of this study include the small number of qualifying runs, the variability in course design associated with a real event, and lack of information about specific faults. The small number of qualifying runs and no information about partial split times limited our ability to make full conclusions, despite a very large event, as dogs only received a calculated speed if they had no faults and were not eliminated. Also, while YPS is a reflection of dog speed as it is calculated based on the course completion time and distance between obstacles, it is only an estimate of average speed. Since UKI measures the shortest distance between obstacles to determine course yardage, the measured distance between obstacles may not accurately reflect the dog’s actual running line between obstacles. The dog’s traveled path is likely longer than the measured distance, and will vary based on size of the dog, speed, training, and handling, among other factors. YPS also only represents the average speed, which does not provide granular information about speed throughout the course, or acceleration and deceleration, all of which could provide valuable information about agility course performance.

With the exception of the 2022 speedstakes course that was run on both grass and dirt, specific individual courses were only run on one substrate. Thus, it is unknown how much the course design contributed to the differences in course performance versus the surface itself. It is also possible that the course design masked some of the surface effects on course performance, and without course design variations the surface effects would have been larger. The relatively smaller variability in calculated speed among standard courses that were all run on dirt both years, may reflect less variability in course design for standard courses or may reflect more similar calculated speeds on a consistent substrate (dirt) compared to jumpers courses. Additionally, as a real event, handlers could choose which events to enter and may have strategically entered some events and not others for a variety of unknown reasons. Likewise, as these events took place shortly after the COVID-19 pandemic, the group of handlers and dogs competing (particularly in 2021) may not fully reflect the population of agility dogs and handlers who would attend such events in future years.

Limitations also included lack of detail about variables within the surface itself, such as specific surface composition, wet versus dry grass, moisture content of the dirt and synthetic sand surfaces, compaction level, environmental humidity levels, and surface maintenance. While the general surface type was provided by the venue, this information was not based on laboratory testing of the surface composition, so exact details of the surface were unknown. We were also not able to assess the environmental factors present throughout the day, such as heat and humidity, both of which not only affect the surface mechanical properties, but also canine exercise physiology. We also could not evaluate associations between faulting of specific obstacles based on surface, time of day or with specific environmental effects. We were unable to assess the effect of surface on the handlers and how that impacted dog speed and performance. Controlled studies will be needed to evaluate these surface and performance variables individually. Despite the numerous limitations, this study provides valuable real-world data from a large number of dogs running the same courses in a random order throughout the day.

### 4.5 Conclusion

Since surface has been demonstrated to contribute to musculoskeletal injury, in both human and equine athletes, it is critical to determine what effects surface has on agility dog biomechanics, performance, and injury. This study provides insight into the complexity of surface effects on performance in agility dogs. It highlights the need for canine-specific surface studies and, in particular, studies on the effect of surface variables on canine agility kinetics and kinematics of performance and how these relate to risk of development of musculoskeletal injuries. Biomechanical and injury studies may help to determine a preferred surface type for agility, both for dog safety and competitiveness. A greater understanding of the complex interactions between surface, biomechanics, and injury is needed to improve the health and longevity of canine agility athletes.

## Data availability statement

The raw data supporting the conclusions of this article will be made available by the authors, without undue reservation.

## Ethics statement

The requirement of ethical approval was waived by The Ohio State University Office of Responsible Research Practices (ORRP) for the studies involving animals because it was a retrospective evaluation of non-sensitive animal data. The studies were conducted in accordance with the local legislation and institutional requirements.

## Author contributions

AP: Conceptualization, Investigation, Methodology, Project administration, Writing – original draft, Writing – review & editing. NK: Investigation, Writing – original draft, Writing – review & editing. LR: Investigation, Writing – original draft, Writing – review & editing. AS: Conceptualization, Data curation, Formal Analysis, Methodology, Writing – original draft, Writing – review & editing.

## References

[1] Pechette Markley A Shoben AB Kieves NR. Internet-based survey of the frequency and types of orthopedic conditions and injuries experienced by dogs competing in agility. J Am Vet Med Assoc. (2021) 259:1001–8. doi: 10.2460/javma.259.9.1001, PMID: 34647477

[2] UK Agility International – Rules and Regulations Ninth Edition (n.d.). Available at: https://ukagilityinternational.com/onewebmedia/UKI%209th%20Edition%20Rule%20Book%20v3.pdf (Accessed June 2, 2024).

[3] Dixon SJ Batt ME Collop AC. Artificial playing surfaces research: a review of medical, engineering and biomechanical aspects. Int J Sports Med. (1999) 20:209–18. doi: 10.1055/s-2007-971119, PMID: 10376475

[4] Nigg BM Yeadon MR. Biomechanical aspects of playing surfaces. J Sports Sci. (1987) 5:117–45. doi: 10.1080/02640418708729771 3326948

[5] Lewis K Northrop AJ Crook GM Mather J Martin JH Holt D. Comparison of equipment used to measure shear properties in equine arena surfaces. Biosyst Eng. (2015) 137:43–54. doi: 10.1016/j.biosystemseng.2015.07.006

[6] Holt D Northrop A Owen A Martin J Hobbs SJ. Use of surface testing devices to identify potential risk factors for synthetic equestrian surfaces. Procedia Eng. (2014) 72:949–54. doi: 10.1016/j.proeng.2014.06.160

[7] Kruse L Traulsen I Krieter J. The use of a technical device for testing the sport-functional properties of riding surfaces. J Equine Vet. (2013) 33:539–46. doi: 10.1016/j.jevs.2012.08.008

[8] Northrop AJ Hobbs SJ Holt D Clayton-Smith E Martin JH. Spatial variation of the physical and biomechanical properties within an equestrian arena surface. Procedia Eng. (2016) 147:866–71. doi: 10.1016/j.proeng.2016.06.288

[9] Peterson ML Wayne McIlwraith C Reiser RF. Development of a system for the in-situ characterisation of thoroughbred horse racing track surfaces. Biosyst Eng. (2008) 101:260–9. doi: 10.1016/j.biosystemseng.2008.07.007

[10] Wannop JW Foreman T Madden R Stefanyshyn D. Influence of the composition of artificial turf on rotational traction and athlete biomechanics. J Sports Sci. (2019) 37:1849–56. doi: 10.1080/02640414.2019.1598923 30922172

[11] Herholz C Siegwart J Nussbaum M Studer MH-P Burgos S. Large temporal variations of functional properties of outdoor equestrian arena surfaces and a new concept of evaluating reactivity with light weight deflectometer settlement curves. J Equine Vet. (2023) 129:104909. doi: 10.1016/j.jevs.2023.104909 37597593

[12] Charalambous L von Lieres und Wilkau HC Potthast W Irwin G. The effects of artificial surface temperature on mechanical properties and player kinematics during landing and acceleration. J Sport Health Sci. (2016) 5:355–60. doi: 10.1016/j.jshs.2015.01.013, PMID: 30356517 PMC6188608

[13] Peterson ML McIlwraith CW. Effect of track maintenance on mechanical properties of a dirt racetrack: a preliminary study. Equine Vet J. (2008) 40:602–5. doi: 10.2746/042516408x330347, PMID: 19031517

[14] Peterson ML Reiser RF Kuo PH Radford DW McIlwraith CW. Effect of temperature on race times on a synthetic surface. Equine Vet J. (2010) 42:351–7. doi: 10.1111/j.2042-3306.2010.00072.x, PMID: 20525055

[15] Ratzlaff MH Hyde ML Hutton DV Rathgeber RA Balch OK. Interrelationships between moisture content of the track, dynamic properties of the track and the locomotor forces exerted by galloping horses. J Equine Vet. (1997) 17:35–42. doi: 10.1016/S0737-0806(97)80456-X

[16] Calderón-Pellegrino G Gallardo L Paredes-Hernández V García-Unanue J Giménez JV Colino E. Influence of artificial turf temperature on physical performance and muscle contractile properties in football players after a repeated-sprint ability test. Sci Rep. (2020) 10:12747. doi: 10.1038/s41598-020-69720-6 32728088 PMC7391762

[17] Gustås P Johnston C Drevemo S. Ground reaction force and hoof deceleration patterns on two different surfaces at the trot. ECP. (2006) 3:209–16. doi: 10.1017/S147806150667607X

[18] Crevier-Denoix N Robin D Pourcelot P Falala S Holden L Estoup P. Ground reaction force and kinematic analysis of limb loading on two different beach sand tracks in harness trotters. Equine Vet J Suppl. (2010) 42:544–51. doi: 10.1111/j.2042-3306.2010.00202.x, PMID: 21059058

[19] Crevier-Denoix N Pourcelot P Ravary B Robin D Falala S Uzel S. Influence of track surface on the equine superficial digital flexor tendon loading in two horses at high speed trot. Equine Vet J. (2009) 41:257–61. doi: 10.2746/042516409x394445, PMID: 19469232

[20] Crevier-Denoix N Falala S Holden-Douilly L Camus M Martino J Ravary-Plumioen B. Comparative kinematic analysis of the leading and trailing forelimbs of horses cantering on a turf and a synthetic surface. Equine Vet J Suppl. (2013) 45:54–61. doi: 10.1111/evj.12160 24304405

[21] Giatsis G Panoutsakopoulos V Kollias IA. Drop jumping on sand is characterized by lower power, higher rate of force development and larger knee joint range of motion. JFMK. (2022) 7:7. doi: 10.3390/jfmk7010017, PMID: 35225903 PMC8883941

[22] Giatsis G Panoutsakopoulos V Frese C Kollias IA. Vertical jump kinetic parameters on sand and rigid surfaces in young female volleyball players with a combined background in indoor and beach volleyball. JFMK. (2023) 8:8. doi: 10.3390/jfmk8030115, PMID: 37606410 PMC10443322

[23] Gaudino P Gaudino C Alberti G Minetti AE. Biomechanics and predicted energetics of sprinting on sand: hints for soccer training. J Sci Med Sport. (2013) 16:271–5. doi: 10.1016/j.jsams.2012.07.003, PMID: 22883597

[24] Northrop AJ Dagg L-A Martin JH Brigden CV Owen AG Blundell EL. The effect of two preparation procedures on an equine arena surface in relation to motion of the hoof and metacarpophalangeal joint. Vet J. (2013) 198:e137–42. doi: 10.1016/j.tvjl.2013.09.048 24360758

[25] Oosterlinck M Royaux E Back W Pille F. A preliminary study on pressure-plate evaluation of forelimb toe-heel and mediolateral hoof balance on a hard vs. a soft surface in sound ponies at the walk and trot. Equine Vet J. (2014) 46:751–5. doi: 10.1111/evj.12210, PMID: 24417416

[26] Openshaw G Northrop AJ Brigden C Martin JH. Preliminary investigation of maximal fetlock extension during jump landing phase on two arena surfaces; two dimensional motion analysis. BSAP Occas Publ. (2006) 35:243–6. doi: 10.1017/S0263967X00042816

[27] Orlande O Hobbs SJ Martin JH Owen AG Northrop AJ. Measuring hoof slip of the leading limb on jump landing over two different equine arena surfaces. Cep. (2012) 8:33–9. doi: 10.3920/CEP11011

[28] Pauchard M Chateau H Camus M Ravary-Plumioen B Falala S Martino J. Effects of speed on the vertical amplitude of the stride in trotters--comparison between fore- and hindlimbs, and influence of the track surface. Comput Methods Biomech Biomed Engin. (2014) 17:144–5. doi: 10.1080/10255842.2014.931555 25074206

[29] Zanetti EM Bignardi C Franceschini G Audenino AL. Amateur football pitches: mechanical properties of the natural ground and of different artificial turf infills and their biomechanical implications. J Sports Sci. (2013) 31:767–78. doi: 10.1080/02640414.2012.750005, PMID: 23230960

[30] Sultan O Nuhmani S Muaidi QI. Comparison of plantar loading patterns on natural grass and artificial turf during various athletic activities. J Sports Med Phys Fitness. (2021) 61:680–6. doi: 10.23736/S0022-4707.21.11342-8 33472347

[31] Mack CD Hershman EB Anderson RB Coughlin MJ McNitt AS Sendor RR. Higher rates of lower extremity injury on synthetic turf compared with natural turf among national football league athletes: epidemiologic confirmation of a biomechanical hypothesis. Am J Sports Med. (2019) 47:189–96. doi: 10.1177/0363546518808499, PMID: 30452873

[32] Kuitunen I Immonen V Pakarinen O Mattila VM Ponkilainen VT. Incidence of football injuries sustained on artificial turf compared to grass and other playing surfaces: a systematic review and meta-analysis. EClinicalMedicine. (2023) 59:101956. doi: 10.1016/j.eclinm.2023.101956, PMID: 37125402 PMC10139885

[33] Gould HP Lostetter SJ Samuelson ER Guyton GP. Lower extremity injury rates on artificial turf versus natural grass playing surfaces: a systematic review. Am J Sports Med. (2023) 51:1615–21. doi: 10.1177/03635465211069562, PMID: 35593739

[34] Murray RC Walters JM Snart H Dyson SJ Parkin TDH. Identification of risk factors for lameness in dressage horses. Vet J. (2010) 184:27–36. doi: 10.1016/j.tvjl.2009.03.020, PMID: 19369100

[35] Moyer W Spencer PA Kallish M. Relative incidence of dorsal metacarpal disease in young thoroughbred racehorses training on two different surfaces. Equine Vet J. (1991) 23:166–8. doi: 10.1111/j.2042-3306.1991.tb02748.x, PMID: 1884696

[36] Tranquille CA Walker VA Roepstorff L Hernlund E Murray RC. Can we use information on the mechanical properties of waxed sand/fibre, sand/fibre and sand/rubber arena surfaces to help understand injury prevention? Equine Vet J. (2013) 45:4–5. doi: 10.1111/evj.12145_10

[37] Georgopoulos SP Parkin TDH. Risk factors for equine fractures in thoroughbred flat racing in North America. Prev Vet Med. (2017) 139:99–104. doi: 10.1016/j.prevetmed.2016.12.006, PMID: 28017453

[38] Balazs GC Pavey GJ Brelin AM Pickett A Keblish DJ Rue J-PH. Risk of anterior cruciate ligament injury in athletes on synthetic playing surfaces: a systematic review. Am J Sports Med. (2015) 43:1798–804. doi: 10.1177/0363546514545864, PMID: 25164575

[39] Sicard GK Short K Manley PA. A survey of injuries at five greyhound racing tracks. J Small Anim Pract. (1999) 40:428–32. doi: 10.1111/j.1748-5827.1999.tb03117.x, PMID: 10516949

[40] Jimenez IA Canapp SO Percival ML. Internet-based survey evaluating the impact of ground substrate on injury and performance in canine agility athletes. Front Vet Sci. (2022) 9:1025331. doi: 10.3389/fvets.2022.1025331, PMID: 36330156 PMC9624126

[41] Classes | UK Agility International (n.d.). Available at: https://ukagilityinternational.com/compete/classes (Accessed March 25, 2024).

[42] Pinnington HC Lloyd DG Besier TF Dawson B. Kinematic and electromyography analysis of submaximal differences running on a firm surface compared with soft, dry sand. Eur J Appl Physiol. (2005) 94:242–53. doi: 10.1007/s00421-005-1323-6, PMID: 15815938

[43] Lejeune TM Willems PA Heglund NC. Mechanics and energetics of human locomotion on sand. J Exp Biol. (1998) 201:2071–80. doi: 10.1242/jeb.201.13.2071, PMID: 9622579

[44] Jafarnezhadgero A Amirzadeh N Fatollahi A Siahkouhian M Oliveira AS Granacher U. Effects of Running on Sand vs. Stable ground on kinetics and muscle activities in individuals with over-pronated feet. Front Physiol. (2021) 12:822024. doi: 10.3389/fphys.2021.822024, PMID: 35095577 PMC8793830

[45] Jafarnezhadgero A Fatollahi A Amirzadeh N Siahkouhian M Granacher U. Ground reaction forces and muscle activity while walking on sand versus stable ground in individuals with pronated feet compared with healthy controls. PLoS One. (2019) 14:e0223219. doi: 10.1371/journal.pone.0223219, PMID: 31557258 PMC6762175

[46] Chateau H Holden L Robin D Falala S Pourcelot P Estoup P. Biomechanical analysis of hoof landing and stride parameters in harness trotter horses running on different tracks of a sand beach (from wet to dry) and on an asphalt road. Equine Vet J Suppl. (2010) 42:488–95. doi: 10.1111/j.2042-3306.2010.00277.x 21059050

[47] Tranquille CA Walker VA Hernlund E Egenvall A Roepstorff L Peterson ML. Effect of superficial harrowing on surface properties of sand with rubber and waxed-sand with fibre riding arena surfaces: a preliminary study. Vet J. (2015) 203:59–64. doi: 10.1016/j.tvjl.2014.10.027, PMID: 25510315

[48] Maeda Y Tomioka M Hanada M Oikawa M. Influence of track surface condition on racing times of thoroughbred racehorses in flat races. J Equine Vet. (2012) 32:689–95. doi: 10.1016/j.jevs.2012.02.012

[49] Arthur RM. Comparison of racing fatality rates on dirt, synthetic, and turf at four California racetracks. Amer Assn Equine. (2010) 56:405–8.

[50] Heinzelmann MM Stokes M Miller SM Bunt SC Hynan LS Didehbani N. Impact of playing surface on concussion symptoms in young American football players. Clin J Sport Med. (2023). doi: 10.1097/JSM.0000000000001204, PMID: 38133559

[51] Rohlf CM Garcia TC Fyhrie DP le Jeune SS Peterson ML Stover SM. Arena surface vertical impact forces vary with surface compaction. Vet J. (2023) 293:105955. doi: 10.1016/j.tvjl.2023.105955, PMID: 36781018

