# Supplementary material for: Evaluation of surface type and time of day on agility course performance
Source: Front Vet Sci. 2024 Jun 26;11:1415634. doi: 10.3389/fvets.2024.1415634 (PMC11233466; doi:10.3389/fvets.2024.1415634)
Supplement: Supplementary file 1 [file Table_1.DOCX]

Supplementary

Supplemental Table 1. Estimated difference in calculated speed difference by order and surface from the 2021 U.S. Open.

|  | Mean difference in speed (YPS, 95% CI) | p-value |
| --- | --- | --- |
| **Dirt** |  |  |
| Jumpers |  |  |
| First rotation | (ref) | 0.24 |
| Second rotation | -0.41 (-0.83, 0.01) |  |
| Third rotation | -0.18 (-0.50, 0.15) |  |
| Fourth rotation | 0.02 (-0.36, 0.40) |  |
| Fifth (last) rotation | 0.03 (-0.39, 0.44) |  |
| Speedstakes |  |  |
| First rotation | (ref) | <0.001 |
| Second rotation | -0.09 (-0.32, 0.14) |  |
| Third rotation | -0.52 (-0.75, -0.30) |  |
| Fourth rotation | -0.35 (-0.56, -0.13) |  |
| Fifth (last) rotation | -0.25 (-0.44, -0.07) |  |
| Standard |  |  |
| First rotation | (ref) | 0.002 |
| Second rotation | 0.14 (-0.06, 0.33) |  |
| Third rotation | 0.21 (-0.02, 0.44) |  |
| Fourth rotation | 0.43 (0.21, 0.65) |  |
| Fifth (last) rotation | 0.27 (0.01, 0.52) |  |
| **Grass** |  |  |
| Jumpers |  |  |
| First rotation | (ref) | 0.44 |
| Second rotation | 0.16 (-0.22, 0.54) |  |
| Third rotation | -0.02 (-0.40, 0.35) |  |
| Fourth rotation | -0.10 (-0.44, 0.24) |  |
| Fifth (last) rotation | 0.16 (-0.17, 0.49) |  |
| **Sand** |  |  |
| Jumpers |  |  |
| First rotation | (ref) | 0.022 |
| Second rotation | 0.05 (-0.25, 0.35) |  |
| Third rotation | -0.05 (-0.28, 0.19) |  |
| Fourth rotation | -0.22 (-0.48, 0.04) |  |
| Fifth (last) rotation | -0.40 (-0.70, -0.10) |  |

Notes: Reported p-values are for the global test of a difference by rotation within each category and are unadjusted for multiple comparisons. YPS = Yards Per Second; negative values indicate slower YPS relative to the reference category. 95% CI = 95% confidence interval, estimated from a model adjusted for height category, if the dog was running select, and accounting for clustering by handler.

Supplemental Table 2. Estimated difference in calculated speed difference by order and surface from the 2022 U.S. Open.

|  | Mean difference in speed (YPS, 95% CI) | p-value |
| --- | --- | --- |
| **Dirt** |  |  |
| Jumpers |  |  |
| First group (1+2) | (ref) | 0.25 |
| Second group (3+4) | -0.17 (-0.34, 0.00) |  |
| Third group (5+6) | -0.08 (-0.26, 0.10) |  |
| Last group (7+8) | -0.11 (-0.28, 0.05) |  |
| Speedstakes |  |  |
| First group (1+2) | (ref) | 0.032 |
| Second group (3+4) | -0.14 (-0.64, 0.37) |  |
| Third group (5+6) | -0.61 (-1.02, -0.20) |  |
| Last group (7+8) | -0.30 (-0.69, 0.10) |  |
| Standard |  |  |
| First group (1+2) | (ref) | 0.049 |
| Second group (3+4) | 0.04 (-0.08, 0.17) |  |
| Third group (5+6) | -0.07 (-0.18, 0.05) |  |
| Last group (7+8) | -0.11 (-0.23, 0.02) |  |
| **Grass** |  |  |
| Jumpers |  |  |
| First group (1+2) | (ref) | 0.25 |
| Second group (3+4) | 0.10 (-0.34, 0.54) |  |
| Third group (5+6) | 0.37 (-0.11, 0.85) |  |
| Last group (7+8) | 0.35 (-0.13, 0.82) |  |
| Speedstakes |  |  |
| First group (1+2) | (ref) | 0.96 |
| Second group (3+4) | 0.09 (-0.39, 0.58) |  |
| Third group (5+6) | 0.03 (-0.52, 0.57) |  |
| Last group (7+8) | 0.12 (-0.42, 0.65) |  |

Notes: Reported p-values are for the global test of a difference by rotation within each category and are unadjusted for multiple comparisons. YPS = Yards Per Second; negative values indicate slower YPS relative to the reference category. 95% CI = 95% confidence interval, estimated from a model adjusted for height category, if the dog was running select, and accounting for clustering by handler.

Supplemental Table 3. Estimated difference in probability of qualifying by order and surface from the 2021 U.S. Open.

|  | Mean difference in probability of qualifying (95% CI) | p-value |
| --- | --- | --- |
| **Dirt** |  |  |
| Jumpers |  |  |
| First rotation | (ref) | 0.50 |
| Second rotation | 0.050 (-0.038, 0.137) |  |
| Third rotation | 0.068 (-0.018, 0.155) |  |
| Fourth rotation | 0.034 (-0.053, 0.121) |  |
| Fifth (last) rotation | 0.007 (-0.073, 0.087) |  |
| Speedstakes |  |  |
| First rotation | (ref) | 0.13 |
| Second rotation | 0.030 (-0.050, 0.109) |  |
| Third rotation | 0.040 (-0.044, 0.123) |  |
| Fourth rotation | 0.092 (0.007, 0.177) |  |
| Fifth (last) rotation | 0.102 (0.007, 0.197) |  |
| Standard |  |  |
| First rotation | (ref) | 0.41 |
| Second rotation | 0.046 (-0.028, 0.119) |  |
| Third rotation | 0.070 (-0.010, 0.150) |  |
| Fourth rotation | 0.014 (-0.055, 0.083) |  |
| Fifth (last) rotation | 0.015 (-0.051, 0.082) |  |
| **Grass** |  |  |
| Jumpers |  |  |
| First rotation | (ref) | 0.17 |
| Second rotation | 0.017 (-0.069, 0.103) |  |
| Third rotation | 0.029 (-0.054, 0.111) |  |
| Fourth rotation | -0.034 (-0.107, 0.040) |  |
| Fifth (last) rotation | 0.064 (-0.019, 0.148) |  |
| **Sand** |  |  |
| Jumpers |  |  |
| First rotation | (ref) | 0.027 |
| Second rotation | 0.088 (0.007, 0.170) |  |
| Third rotation | 0.118 (0.037, 0.200) |  |
| Fourth rotation | 0.036 (-0.040, 0.112) |  |
| Fifth (last) rotation | 0.077 (0.001, 0.153) |  |

Notes: Positive differences in probability of qualifying indicate larger probability of qualifying relative to the reference category. Reported p-values are for the global test of a difference by rotation within each category and are unadjusted for multiple comparisons. 95% CI = 95% confidence interval, estimated from a model adjusted for height category, if the dog was running select, and accounting for clustering by handler.

Supplemental Table 4. Estimated difference in probability of qualifying by order and surface from the 2022 U.S. Open.

|  | Mean difference in probability of qualifying (95% CI) | p-value |
| --- | --- | --- |
| **Dirt** |  |  |
| Jumpers |  |  |
| First group (1+2) | (ref) | 0.15 |
| Second group (3+4) | 0.068 (-0.001, 0.137) |  |
| Third group (5+6) | 0.044 (-0.021, 0.109) |  |
| Last group (7+8) | 0.064 (0.000, 0.128) |  |
| Speedstakes |  |  |
| First group (1+2) | (ref) | 0.89 |
| Second group (3+4) | 0.028 (-0.121, 0.177) |  |
| Third group (5+6) | 0.007 (-0.132, 0.147) |  |
| Last group (7+8) | 0.052 (-0.089, 0.193) |  |
| Standard |  |  |
| First group (1+2) | (ref) | 0.082 |
| Second group (3+4) | -0.033 (-0.072, 0.006) |  |
| Third group (5+6) | 0.015 (-0.027, 0.058) |  |
| Last group (7+8) | 0.010 (-0.031, 0.050) |  |
| **Grass** |  |  |
| Jumpers |  |  |
| First group (1+2) | (ref) | 0.32 |
| Second group (3+4) | 0.056 (-0.018, 0.130) |  |
| Third group (5+6) | 0.041 (-0.036, 0.118) |  |
| Last group (7+8) | -0.001 (-0.069, 0.068) |  |
| Speedstakes |  |  |
| First group (1+2) | (ref) | 0.83 |
| Second group (3+4) | 0.030 (-0.049, 0.109) |  |
| Third group (5+6) | 0.034 (-0.043, 0.111) |  |
| Last group (7+8) | 0.021 (-0.060, 0.103) |  |

Notes: Positive differences in probability of qualifying indicate larger probability of qualifying relative to the reference category. Reported p-values are for the global test of a difference by rotation within each category and are unadjusted for multiple comparisons. 95% CI = 95% confidence interval, estimated from a model adjusted for height category, if the dog was running select, and accounting for clustering by handler.
